# A Phase Ib dose-escalation study to evaluate safety and tolerability of the addition of the aminopeptidase inhibitor tosedostat (CHR-2797) to paclitaxel in patients with advanced solid tumours

**DOI:** 10.1038/sj.bjc.6605917

**Published:** 2010-09-28

**Authors:** C M L van Herpen, F A L M Eskens, M de Jonge, I Desar, L Hooftman, E A Bone, J N H Timmer-Bonte, J Verweij

**Affiliations:** 1 Department of Medical Oncology, Radboud University Nijmegen Medical Centre, PO Box 9101, Nijmegen 6500 HB, The Netherlands; 2 Department of Medical Oncology, Erasmus University Medical Center, Rotterdam, The Netherlands; 3 Chroma Therapeutics Ltd., 93 Milton Park, Abingdon OX14 4RY, UK

**Keywords:** Phase I, aminopeptidase inhibitor, CHR-2797, tosedostat, paclitaxel

## Abstract

**Background:**

This Phase Ib dose-escalating study investigated safety, maximum tolerated dose (MTD), dose-limiting toxicity (DLT), pharmacokinetics (PK) and clinical antitumour activity of tosedostat (CHR-2797), an orally bioavailable aminopeptidase inhibitor, in combination with paclitaxel.

**Methods:**

A total of 22 patients received paclitaxel (135–175 mg m^−2^) intravenously, administered once every three weeks for up to six cycles, with oral tosedostat (90–240 mg) daily.

**Results:**

One DLT (grade 3 dyspnoea) was observed in one patient with tosedostat 180 mg combined with paclitaxel 175 mg m^−2^. A high number of paclitaxel infusion reactions was noted during the second administration (59%) and this prompted interruption of tosedostat dosing for 5 days around every second and subsequent paclitaxel infusion. No formal MTD was determined because of the high frequency of paclitaxel infusion reactions that may have been influenced by tosedostat. Most frequently observed drug-related adverse events were alopecia, fatigue (95% each), peripheral sensory neuropathy (59%), paclitaxel hypersensitivity (59%) and rash (55%). One patient died because of eosinophilic myocarditis, possibly related to study medication. There was no PK interaction between tosedostat and paclitaxel. In all, 3 patients had a partial response and 12 patients had stable disease lasting >3 months.

**Conclusion:**

The combination of tosedostat with paclitaxel was well tolerated except for the high incidence of paclitaxel-related infusion reactions.

Tosedostat (CHR-2797) is a novel metalloenzyme inhibitor that is converted intracellularly into a pharmacologically active metabolite CHR-79888. Being a poorly membrane-permeant acid, intracellular accumulation of CHR-79888 is excellent. Tosedostat is both antiproliferative and proapoptotic, and has demonstrated antiangiogenic effects. Both *in vitro* and *in vivo* experiments have shown selectivity for transformed over nontransformed cells (Krige *et al*, 2008). CHR-79888 is a potent inhibitor of various intracellular aminopeptidases, a number of which are overexpressed in certain human tumour types. Aminopeptidases catalyse the sequential removal of amino acids from the amino-terminus of peptide/protein substrates, thereby regulating the function of biologically active peptides, trimming antigens for MHC class 1 presentation and modulating protein recycling (Saric *et al*, 2004).

Although the mechanism of the antiproliferative effect of aminopeptidase inhibition remains to be fully elucidated, gene expression analysis of the human promyelocytic leukaemia cell line HL-60, exposed to tosedostat revealed a transcriptional response to the drug indicative of amino acid depletion, a so-called amino acid deprivation response (AADR; Krige *et al*, 2008). Tosedostat also inhibited phosphorylation of mTOR substrates and reduced protein synthesis in these cells, indicating amino acid depletion (Krige *et al*, 2008). One of the consequences of AADR is upregulation of proapoptotic protein markers such as CHOP (C/EBP homologous protein) and Noxa. Taking these data together suggests that tosedostat depletes sensitive tumour cells of amino acids by blocking protein recycling and thereby generates an antiproliferative effect (Figure 1). Tosedostat synergises with a wide range of chemotherapeutic agents in inducing antiproliferative effects in a wide range of cancer cell lines *in vitro* (Jenkins *et al*, 2007; Moore *et al*, 2009).

Here, we present results of a Phase Ib trial (EudraCT number 2006–002498–35) designed to determine maximum tolerated dose (MTD), dose-limiting toxicities (DLTs), pharmacokinetics (PK) and preliminary activity of the combination of continuous (once) daily tosedostat dosing, and 3-weekly paclitaxel infusions.

## Patients and methods

### Patient eligibility

Eligible patients were aged ⩾18 years, and had histologically or cytologically confirmed advanced solid malignancies, refractory to conventional treatment. Patients were also required to have life expectancy ⩾12 weeks, Eastern Cooperative Oncology Group (ECOG) performance status ⩾2, adequate haematopoietic (absolute neutrophil count ⩾1.5 × 10^9^ l^−1^; platelets ⩾100 × 10^9^ l^−1^), hepatic (bilirubin ⩽1.5 × upper normal limit (ULN), aspartate transaminase/alanine transaminase ⩽2.5 °C ULN) and renal (creatinine ⩾1.5 × ULN) function. Patients with previous anticancer therapy within 4 weeks of study entry (6 weeks for mitomycin and nitrosureas), known brain tumours or brain metastases and patients who failed to recover from acute adverse effects of previous therapies or who had received more than four previous chemotherapy regimens were excluded. The local ethics committees at both participating centres approved the study protocol and written informed consent was obtained from all patients before any study-related procedures.

### Study design and dose-escalation schedule

Cohorts of three to six patients were administered intravenous (i.v.) paclitaxel over 3 h every 21 days in combination with escalating oral doses of tosedostat.

Patients received up to six cycles of paclitaxel. Premedication consisted of dexamethasone, clemastine and a histamine H_2_-receptor antagonist and was administered i.v. 30–60 min before paclitaxel. Tosedostat capsules (10, 20 and 40 mg) were taken after food at the same time every day from day 2 onwards, with the exception of day 22, when blood was drawn for a second PK profile and tosedostat was withheld until 1 h after the end of the paclitaxel infusion.

The first cohort of three patients received a low, but registered and effective dose of paclitaxel (135 mg m^−2^). The starting dose of CHR-2797 was 90 mg daily, below the MTD. Other planned cohorts in this study were: *cohort 2*: paclitaxel 175 mg m^−2^ and tosedostat 90 mg; *cohort 3*: paclitaxel 175 mg m^−2^ and tosedostat 130 mg; *cohort 4*: paclitaxel 175 mg m^−2^ and tosedostat 180 mg; *cohort 5*: paclitaxel 175 mg m^−2^ and tosedostat 240 mg; *cohort 6*: paclitaxel 200 mg m^−2^ and tosedostat 240 mg. After *cohort 4*, an amendment was implemented allowing for dose interruption of tosedostat, which resulted in the following cohorts: *cohort 5*: paclitaxel 175 mg m^−2^ and tosedostat 180 mg from day 2–17 of each cycle; *cohort 6*: paclitaxel 175 mg m^−2^ and tosedostat 240 mg from day 2–17 of each cycle.

Patients remained on therapy for as long as the investigator felt that it was in their best interest and while there was no evidence of progressive disease (PD) or unacceptable toxicity. Following completion of paclitaxel therapy, patients could continue with single agent tosedostat until evidence of PD or unacceptable toxicity.

### Definition of MTD and DLT

Toxicity was evaluated according to common toxicity criteria for adverse events (CTCAEv3.0). The MTD was defined as the dose level(s) at which at least two out of six patients developed DLT. This was defined as any of the following events possibly or probably related to the paclitaxel/tosedostat combination and which occurred during the first 21 days of treatment: grade 4 neutropenia lasting ⩾7 days or neutropenic fever/sepsis; grade 4 thrombocytopenia; any drug-related, nonhaematological grade 3–4 toxicity with the exceptions of fatigue and inadequately treated nausea and vomiting; a delay in retreatment with paclitaxel of >7 days.

### Patient evaluation and follow-up

Toxicity assessment, haematology and clinical biochemistry were performed at baseline and weekly during the study. Physical and ECOG performance status were recorded at baseline and before the next cycle.

Response was evaluated according to Response Evaluation Criteria in Solid Tumors (Therasse *et al*, 2000) after every second cycle.

### PK assessments

Pharmacokinetic samples were taken on days 1, 21 and 22, with a 24 h sample taken the following day, for determination of plasma PK profiles of paclitaxel, tosedostat and CHR-79888. Subsequent to dose interruptions permitted by amendment 2, it was no longer meaningful to obtain full PK profiles, so sampling in cohorts 5 and 6 was reduced to one sample, taken before paclitaxel infusion on day 22, for the determination of trough concentrations of tosedostat and CHR-79888 in plasma. Plasma concentrations of tosedostat, CHR-79888 and paclitaxel were measured using validated LC-MS/MS bioanalytical methods.

The effect of tosedostat coadministration on the PK of paclitaxel was evaluated by comparing PK parameters from the infusion of day 1 with those of day 22. The effect of paclitaxel on the PK of tosedostat and CHR-79888 was evaluated by comparing PK parameters of day 21 with those of day 22 (the day of second paclitaxel infusion). On day 21, samples were taken until 8 h post-dose; the day 22 predose sample was used as the 24 h sample of day 21. Samples were taken until 24 h after the day 22 dose of tosedostat. Peak plasma concentrations (*C*_max_), overall drug exposure (AUC), and terminal plasma half-life (*t*_½_) were calculated using noncompartmental methods using WinNonlin Professional software (version 4.1, Pharsight Corporation, St Louis, MO, USA). Pharmacokinetics analysis, with reference to possible interactions, was descriptive.

## Results

### General trial conduct

This study was conducted at two academic cancer centres between August 2006 and November 2007. In total, 22 patients were enrolled. Patient characteristics are summarised in Table 1. One patient was withdrawn after 7 days of treatment because of early PD and was replaced; consequently, 21 patients were evaluable for efficacy analyses, all of whom received at least two treatment cycles. Six patients received just two cycles, one patient received three cycles, five patients received four cycles, two patients received five cycles and seven patients received six cycles. There was no apparent correlation between number of cycles and dose levels. Seven continued on tosedostat monotherapy: six patients had completed six cycles of paclitaxel therapy and in one patient paclitaxel was stopped after two infusions due to sensory neuropathy.

### DLTs and MTD

One patient with urethral cancer treated in cohort 5 (175 mg m^−2^ paclitaxel, 180 mg tosedostat) experienced DLT: CTC grade 3 dyspnoea, with grade 2 fever and persistent grade 3 urinary tract infection. In this patient, tosedostat was reduced to 130 mg and subsequently this cohort was expanded with three additional patients, none of whom developed DLT.

There were no further DLTs in this trial. The three patients in cohort 6 (175 mg m^−2^ paclitaxel, 240 mg tosedostat) completed the dose-escalation phase without any grade 3/4 toxicity. Nevertheless, the trial steering committee decided to terminate the study. Formal MTD was never reached in this trial, but in cohorts 3–6 paclitaxel infusion reactions occurred in 73% of patients, despite routine premedication.

### Overall safety and tolerability

#### Adverse events (AEs) and serious adverse events (SAEs).

All patients experienced one or more AEs. The majority of these AEs were disease-related and/or known side effects of paclitaxel and were less often considered tosedostat-related by the investigators. Table 2 summarises AEs occurring with a frequency of >20% or grade ⩾3 in cycle 1 and in all cycles. The most frequently reported AEs were alopecia (95%), fatigue (95%), peripheral sensory neuropathy (59%), rash (55%) and drug hypersensitivity reaction (HSR; 50%), which with interruptions of the paclitaxel infusion and individually reported symptoms, contributed to an overall 59% incidence of infusion reactions.

A total of 19 SAEs were reported in 12 patients. In six patients SAEs were considered paclitaxel and/or tosedostat-related. These were decreased fluid intake (*n*=1), allergic reaction (*n*=1), dyspnoea (*n*=1), eosinophilic myocarditis (*n*=1) and renal insufficiency (*n*=2). In all, 13 SAEs were considered disease-related.

One patient (in Cohort 5) died 6 days after his third paclitaxel infusion and 2 days after his last dose of tosedostat. He had been a professional body builder for many years and his lifestyle included a diet of up to 30 eggs per day in preparation for competitions and the intermittent use of anabolic steroids (this was confirmed after his death by relatives). An initial diagnosis of chondrosarcoma was made in 2005. His medical history included hypertension, chronic obstructive pulmonary disease and atypical retrosternal chest pain (for several years), thought to be related to a hiatus hernia. His pretreatment ECG had shown marked ST-T wave abnormalities with signs of a possible old myocardial infarction (MI). After 4 days of his third paclitaxel infusion, he was admitted to hospital as an emergency with an exacerbation of chest pain suggestive of MI. Tosedostat was discontinued. After 2 days, he died from cardiac failure with ventricular fibrillation and electromechanical dissociation. A post-mortem examination revealed a dilated concentric cardiomyopathy with hypertrophy of both ventricles (weight of heart 530 g, compared with ∼350 g for a normal heart), probably of chronic nature. An expert cardiac pathologist reviewed slides of the myocardial tissue. Dense interstitial lymphocytic and eosinophilic infiltrates throughout the ventricles were observed. Other findings were a concomitant eosinophilic infiltrate in the liver and signs of incomplete suppression of peripheral eosinophils, despite an apparent systemic stress response. Consequently, the cause of death was eosinophilic myocarditis, considered possibly related to paclitaxel, tosedostat or other medications.

One patient in cohort 5 discontinued paclitaxel after two cycles following development of grade 3 sensory neuropathy. This patient had a history of diabetes mellitus and metastatic colorectal cancer, for which he had received previous systemic treatment including oxaliplatin, capecitabine, bevacizumab, cetuximab and irinotecan. During the first cycle he developed sensory neuropathy grade 1, which increased to grade 3 after the second cycle. Neuropathy was considered possibly related to tosedostat and definitely related to paclitaxel. The patient continued with tosedostat monotherapy for 7 weeks until PD. The neuropathy did not resolve. Neuropathy led to delay in dosing or dose reduction of paclitaxel in four other patients and tosedostat dose interruption in one patient.

#### Paclitaxel infusion reactions.

Infusion-related HSRs (grade 1, three patients; grade 2, one patient; grade 3, seven patients) or infusion interruptions (two patients, symptoms consistent with such reactions) were reported in 59% of patients during second and/or subsequent paclitaxel administrations. They are summarised per dose level in Table 3. Before cohort 3, the paclitaxel infusion schedule was amended to accommodate PK sampling alongside the infusion interruption and additional premedication required to manage these reactions. Before cohort 5, the regimen was further modified by interrupting tosedostat dosing from 4 days before to 1 day after each paclitaxel infusion. This did reduce incidence and severity of HSRs to some extent in cohort 5, but in cohort 6 all patients experienced HSRs at their second paclitaxel administration. All HSRs could be controlled medically.

#### Laboratory parameters.

For the main haematology parameters, except for APTT, median values dropped after the first and subsequent paclitaxel infusions, reaching a nadir on day 8 (platelets) or day 15 (haemoglobin, WBC, neutrophils and lymphocytes) of each cycle. There was recovery to baseline value (lymphocytes) or below baseline (other parameters) on day 21. In subsequent cycles, WBC and neutrophil counts also tended to recover to baseline values, whereas lymphocyte counts showed a rebound increase to above baseline values by day 21 of cycles 4 and 5. Median platelet count and haemoglobin values did not recover to baseline values during any of the cycles. Other differential counts were recorded, but no changes of interest were observed.

### PK

The overall exposure to tosedostat and CHR-79888 increased in a dose proportional manner.

#### Effect of coadministration of paclitaxel on PK of tosedostat and CHR-79888.

The effect of coadministration of paclitaxel on PK of tosedostat and CHR-79888 was evaluated by comparing PK parameters of days 21 and 22. Overall exposure to tosedostat was unaffected by paclitaxel administration. However, a tendency for a decreased *C*_max_ and an increased *t*_max_ and *t*_½_ was observed, suggesting that coadministration of paclitaxel affected the shape of the tosedostat PK profile, but not the overall exposure. There was no significant effect of paclitaxel on *C*_max_, AUC_0−*t*_, *t*_max_ and *t*_½_ values for CHR-79888 (Table 4).

#### Effect of coadministration of tosedostat on the PK of paclitaxel.

The effect of tosedostat on PK of paclitaxel was evaluated by comparing PK parameters of paclitaxel of days 1 and 22. The PK profiles were essentially overlapping (data not shown).

### Antitumour activity

Partial responses (PR) were observed in 3 patients with malignant melanoma, squamous cell non-small-cell lung cancer and squamous-cell carcinoma of the oesophagus (14% all confirmed after ⩾4 weeks) and stable disease was observed in 12 patients (57%). The three PRs occurred at various dose levels and response durations were 7.2, 7.1 and 1.5 months, respectively. Median (95% CI) duration of s.d. was 5.6 (3–6.5) months.

## Discussion

The development of drugs that elicit an antiproliferative effect by blocking intracellular protein recycling in transformed cells represents a novel approach to the treatment of solid tumours and haematological malignancies. The novel aminopeptidase inhibitor tosedostat causes an AADR in malignant cells and also inhibits angiogenesis; both effects may exert additional antitumour activity when given in combination with chemotherapy.

The safety profile of oral daily dosing with tosedostat in a single agent Phase I setting has been reported previously (Reid *et al*, 2009) and found to be good, with fatigue, thrombocytopenia, peripheral oedema and diarrhoea as the most commonly reported AEs; MTD with single agent tosedostat in solid tumour patients treated for at least 28 days was 240 mg. Dose-limiting toxicities were reported in two of four patients treated at 320 mg because of a combination of thrombocytopenia, dizziness and visual abnormalities in one patient, and anaemia, blurred vision and vomiting in a second patient, leading to the patients being unable to complete 28 days of daily oral therapy.

This Phase 1b dose-escalation study was designed to investigate the clinical safety, PK and preliminary antitumour activity of daily oral tosedostat when administered with 3-weekly paclitaxel in patients with advanced or metastatic cancer. Maximum tolerated dose was not reached in this study. Apart from the infusion reactions, combined tosedostat and paclitaxel therapy was well tolerated, with only one DLT observed in 22 patients. AEs were rarely more than moderate and were easily managed. The incidence and severity of the main acute toxic effects of neutropenia/leukopenia, anaemia, myalgia and nausea/vomiting were not increased relative to paclitaxel alone.

A total of 13 patients (59%) experienced symptoms consistent with an infusion reaction to paclitaxel, despite a routinely given prophylactic regimen of dexamethasone plus histamine-1 and -2 receptor antagonists. One of the major limitations associated with the use of paclitaxel and its Cremophor EL formulation concerns HSRs. The mechanism of paclitaxel HSRs is not entirely known. Cremophor EL is suspected to be the allergen (Weiss *et al*, 1990), but complement and mast cell activation may be involved. Premedication regimens and longer infusion times reduced reactivity to paclitaxel in the 1990s, although in the presence of premedication this phenomenon continues to occur in 10–34% of patients (Bristol Myers Squibb (SPC), 2005). Although the HSRs can be medically managed, they can be of considerable concern to patients (Markham *et al*, 2000). Typically, around half of these reactions occur during the initial infusion, but all HSRs in our combination trial were reported during second and subsequent paclitaxel infusions. In an attempt to reduce the possible stimulatory effect of tosedostat on paclitaxel-induced HSRs, and taking into consideration the plasma *t*_½_ of CHR-79888 of 6–11 h, it was decided to introduce a 5-day dosing window around second and subsequent paclitaxel infusions in cohort 5. Although this appeared to have a positive effect in patients on trial at that time (two out of six patients experienced a HSR), all three patients in the next cohort developed a HSR. Patients in cohorts 5 and 6 received the same dose of paclitaxel (175 mg m^−2^), but the dose of tosedostat was increased from 180 to 240 mg. Even though paclitaxel-related HSR was not included in the DLT definitions, the investigators attributed the higher incidence of HSR to the combination of tosedostat and paclitaxel; consequently, it was decided not to proceed with a planned dose escalation of paclitaxel to 200 mg m^−2^. Because tosedostat had also reached the MTD as determined in the single agent Phase I study, further dose escalations were not indicated (Reid *et al*, 2009). A formal explanation as to how tosedostat could enhance HSR is lacking, but immunostimulatory activity has been described with the use of the aminopeptidase inhibitor bestatin (Talmadge *et al*, 1986; Ichinose *et al*, 2003). It is probable that these infusion-related reactions could be avoided by the use of a cremophor-free formulation of paclitaxel (Gradishar, 2006).

In the patient who died during the study, a possible relationship between this fatality and study drugs could not be excluded. We attempted to identify the aetiology of the confirmed eosinophilic myocarditis. Clearly, drugs scored high amongst the possible candidates (Ginsberg and Parrillo, 2005), but in this patient there was also a previous medical history of retrosternal pains, and his pretreatment ECG revealed signs of cardiomegaly.

Tosedostat has been associated with a platelet suppressive effect in the single agent dose escalation studies (Ossenkoppele *et al*, 2009; Reid *et al*, 2009). Although this did not require dose interruption in patients treated with tosedostat monotherapy, this may have been responsible for the delayed recovery after every paclitaxel infusion in this combination study. Otherwise, the cyclical pattern observed for the haematology parameters, with a drop in values after each paclitaxel infusion that reached a nadir on day 8 or day 15 of each cycle and recovered to baseline or just below baseline on day 21, suggests that the observed phenomenon was paclitaxel-related, although an additive effect of tosedostat cannot be ruled out.

When tosedostat was coadministered with paclitaxel, the exposure to tosedostat, as measured by the AUC_0−*t*_, seemed to have been unaffected by paclitaxel coadministration, although the shape of the tosedostat profile may have been affected in some patients. There was no observable effect of coadministration of paclitaxel on the PK of CHR-79888. When paclitaxel was coadministered with tosedostat, the PK of paclitaxel seemed to be unaffected.

Treatment successes in early phase studies with tosedostat monotherapy included a PR and several patients with disease stabilisation of at least 6 months' duration in patients with metastatic cancer, and a 31.4% response rate in patients with relapsed/refractory AML (Ossenkoppele *et al*, 2009; Reid *et al*, 2009). In this combination study of 21 assessable patients with relapsed, heavily pretreated solid tumours, 3 (14%) had a PR. It is not possible to determine whether the responses seen in this study were induced by paclitaxel alone or whether the addition of tosedostat contributed to these effects; however, this response rate seemed similar to taxane monotherapy.

In conclusion, except for the high incidence of paclitaxel-related infusion reactions despite the use of routine prophylactic regimes, the combination of tosedostat with paclitaxel was well tolerated. As PK parameters of paclitaxel appeared very similar when given alone or in the presence of tosedostat, increased exposure to paclitaxel cannot be the explanation for this increased incidence. Treatment with this combination and regimen was considered to be essentially safe, however, further development of tosedostat administered with cremophor-formulated paclitaxel cannot be recommended. The antiproliferative, synergistic and potential immuno-modulatory properties of tosedostat do, however, warrant further exploration in studies with cremophor-free formulations of paclitaxel and with other agents.

## Figures and Tables

**Figure 1:**
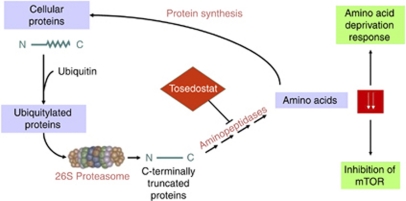
Mechanism of action of tosedostat. Tosedostat inhibits aminopeptidase activity, which results in the depletion of cellular amino acid pools selectively in tumour cells. This disrupts the turnover of cell cycle intermediates in such a way that it impacts cancer cell survival or proliferation.

**Table 1:** Patient characteristics (*n*=22)

Age, median (range; years)	59 (34–72)
Male/female	17/5
	
*Performance score*	
0	12
1	8
2	2
	
*Tumour type*	
Sarcoma	4
Bladder cancer	3
Gastric cancer	3
Colorectal cancer	2
NSCLC	2
Miscellaneous	8
	
*Previous treatment*	
Systemic treatment (alone or in combination)	19
Chemotherapy	19
• 1 regimen	11
• 2 regimens	6
• 3 and 4 regimens	2
Docetaxel	2
Tyrosine kinase inhibitors	4
Hormonal treatment	1
Surgery (alone or in combination)	17
Radiotherapy (with other treatments)	10

Abbreviation: NSCLC=non-small-cell lung cancer.

**Table 2:** Adverse events occurring in more than 20% of patients or any grade 3–5 during the first 21 days and during all cycles of treatment, worst grade per person and regardless of relationship

	**Incidence of AEs during first 21 days, worst grade per patient regardless of relation**							**Incidence of AEs during all cycles, worst grade per patient regardless of relation**						
	**CTCAE grade**					**No. of patients with events**		**CTCAE grade**					**No. of patients with events**	
**Adverse event**	**1**	**2**	**3**	**4**	**5**	***n***	**%**	**1**	**2**	**3**	**4**	**5**	***n***	**%**
*Blood and lymphatic system disorders*														
Anaemia	1	1	0	0	0	2	9	1	3	1	0	0	5	23
Leukopenia	0	2	2	0	0	4	18	0	1	3	0	0	4	18
Neutropenia	0	1	2	3	0	6	27	0	0	2	4	0	6	27
														
*Cardiac disorders*														
Eosinophilic myocarditis	0	0	0	0	0	0	0	0	0	0	0	1	1	5
														
*Gastrointestinal disorders*														
Constipation	3	1	0	0	0	4	18	3	3	0	0	0	6	27
Diarrhoea	2	0	0	0	0	2	9	5	3	0	0	0	8	36
Gastric dilatation	0	0	1	0	0	1	5	0	0	1	0	0	1	5
Nausea	4	1	0	0	0	5	23	9	1	0	0	0	10	45
Vomiting	3	1	0	0	0	4	18	3	2	0	0	0	5	23
														
*General disorders and administration site conditions*														
Fatigue	7	2	1	0	0	10	45	5	10	6	0	0	21	95
General physical health deterioration	0	0	0	0	0	0	0	0	0	1	0	0	1	5
Malaise		0	1	0	0	1	5	1	1	1	0	0	3	14
Mucosal inflammation	2	1	0	0	0	3	14	4	1	0	0	0	5	23
Oedema	3	0	0	0	0	3	14	4	0	1	0	0	5	23
Oedema peripheral	4	1	0	0	0	5	23	6	2	0	0	0	8	36
Pyrexia	1	1	0	0	0	2	9	4	2	0	0	0	6	27
														
*Immune system disorders*														
Drug hypersensitivity	0	0	0	0	0	0	0	3	1	7	0	0	11	50
														
*Infections and infestations*														
Bronchitis	0	0	0	0	0	0	0	0	0	1	0	0	1	5
Cystitis	0	0	0	0	0	0	0	0	1	1	0	0	2	9
Nasopharyngitis	2	0	0	0	0	2	9	6	0	0	0	0	6	27
Urinary tract infection	0	0	1	0	0	1	5	0	1	1	0	0	2	9
														
*Investigations*														
Alanine aminotransferase increased	1	2	0	0	0	3	14	1	1	1	0	0	3	14
Blood bilirubin	0	0	0	0	0	0	0	0	0	1	0	0	1	5
γ-glutamyltransferase increased	0	0	1	0	0	1	5	0	1	1	0	0	2	9
														
*Metabolism and nutrition disorders*														
Anorexia	4	0	0	0	0	4	18	6	2	2	0	0	10	45
Dehydration	0	0	1	0	0	1	5	0	1	1	0	0	2	9
Diabetes mellitus	0	0	1	0	0	1	5	0	0	1	0	0	1	5
Hypercalcaemia	0	0	0	0	0	0	0	0	0	1	0	0	1	5
Hyperglycaemia	0	0	2	0	0	2	9	0	1	2	0	0	3	14
														
*Musculoskeletal and connective tissue disorders*														
Arthralgia	5	0	0	0	0	5	23	5	0	0	0	0	5	23
Back pain	2	0	0	0	0	2	9	3	2	0	0	0	5	23
Myalgia	7	1	1	0	0	9	41	8	1	1	0	0	10	45
														
*Neoplasms benign, malignant and unspecified (including cysts and polyps)*														14
Tumour pain	0	0	1	0	0	1	5	0	1	2	0	0	3	14
														
*Nervous system disorders*														95
Dizziness	4	1	0	0	0	5	23	8	1	1	0	0	10	45
Peripheral sensory neuropathy	4	3	0	0	0	7	32	6	4	3	0	0	13	59
Spinal cord compression	0	0	0	0	0	0	0	0	0	1	0	0	1	5
														
*Renal and urinary disorders*														23
Pollakiuria	0	0	0	0	0	0	0	0	0	1	0	0	1	5
														
*Respiratory, thoracic and mediastinal disorders*														73
Cough	5	0	0	0	0	5	23	8	1	0	0	0	9	41
Dyspnoea	0	0	1	0	0	1	5	6	1	3	0	0	10	45
Pulmonary embolism	0	0	0	0	0	0	0	0	0	1	0	0	1	5
														
*Skin and subcutaneous tissue disorders*														100
Alopecia	7	8	0	0	0	15	68	1	20	0	0	0	21	95
Dry skin	0	0	1	0	0	1	5	3	0	1	0	0	4	18
Erythema	0	0	0	0	0	0	0	1	0	1	0	0	2	9
Hyperhidrosis	1	0	0	0	0	1	5	2	0	1	0	0	3	14
Pruritus	3	0	1	0	0	4	18	5	0	1	0	0	6	27
Rash	3	1	1	0	0	5	23	9	1	2	0	0	12	55

Abbreviations: AEs=adverse events; CTCAE=common toxicity criteria for adverse events.

**Table 3:** Incidence of hypersensitivity reactions per dose level in cycle 2

**Cohort**	**Patients with hypersensitivity reaction/patients treated in cohort**
1	2/3
2	0/4^a^
3	3/3
4	3/3
5	2/6
6	3/3

a Including Patient 4907 who was withdrawn after 7 days study treatment because of early progressive disease.

**Table 4:** Effect of the coadministration of paclitaxel on the PK parameters (mean values (±s.e.m.)) of tosedostat (CHR-2797) and CHR-79888 (day 21 tosedostat without paclitaxel; day 22 tosedostat in combination with paclitaxel)

**Cohort/doses**	**Analyte**	**Day**	**Cmax (ng ml^−1^)**	**Tmax (h)**	**AUC_0−t_ (ng h ml^−1^)**	**T_½_ (h)**	**V_z_/F**	**Cl/F**
1. CHR-2797 90 mg	CHR-2797	21	522 (165)	1.2 (0.4)	1140 (401)	1.2 (0.1)	179 (67)	1771 (709)
Paclitaxel 135 mg m^−2^		22	289 (124)	4.0 (2.0)	896 (266)	2.4 (1.4)	258 (91)	1556 (514)
	CHR-79888	21	544 (56)	4.7 (0.7)	6250 (311)	6.1 (0.2)	116 (9)	221 (11)
		22	500 (108)	6.0 (1.2)	5550 (1680)	8.6 (ND)	103 (ND)	139 (ND)
								
2. CHR-2797 90 mg	CHR-2797	21	969 (367)	1.2 (0.4)	1890 (382)	1.3 (0.2)	72 (12)	654 (14)
Paclitaxel 175 mg m^−2^		22	527 (119)	2.0 (0)	1410 (315)	3.1 (1.0)	276 (84)	1160 (256)
	CHR-79888	21	324 (40)	5.3 (0.7)	3510 (519)	5.6 (0.2)	204 (38)	417 (65)
		22	364 (16)	4.7 (0.7)	4570 (438)	7.5 (1.0)	181 (7)	290 (37)
								
3. CHR-2797 130 mg	CHR-2797	21	880 (87)	1.7 (0.3)	1920 (328)	1.1 (0)	112 (22)	1204 (249)
Paclitaxel 175 mg m^−2^		22	615 (225)	1.3 (0.3)	1400 (457)	4.0 (2.7)	587 (296)	2011 (833)
	CHR-79888	21	872 (37)	3.3 (0.7)	9440 (892)	5.9 (0.5)	108 (4)	217 (27)
		22	723 (101)	5.3 (0.7)	9340 (1530)	7.1 (1.1)	142 (22)	242 (73)
								
4. CHR-2797 180 mg	CHR-2797	21	1400 (550)	1.7 (0.3)	2520 (730)	1.1 (0.1)	123 (26)	1352 (304)
Paclitaxel 175 mg m^−2^		22	399 (80)	4.0 (0)	1710 (99)	3.1 (0.3)	471 (70)	1758 (96)
	CHR-79888	21	1270 (240)	4.0 (0)	9590 (1090)	4.6 (0)	113 (14)	283 (37)
		22	1030 (188)	5.3 (0.7)	11600 (1620)	6.2 (0.3)	130 (19)	242 (30)

Abbreviations: AUC_0−*t*_=area under the curve; Cl/F=Apparent plasma clearance; Cmax=maximum plasma concentration; ND=not determined; PK=pharmacokinetics; Tmax=time of Cmax; *t*_½_=biological half time; V_z_/F=apparent volume of distribution.
